# Synthesis of Chemically Modified Acid-Functionalized Multiwall Carbon Nanotubes with Benzimidazole for Removal of Lead and Cadmium Ions from Wastewater

**DOI:** 10.3390/polym15061421

**Published:** 2023-03-13

**Authors:** Ibrahim Elghamry, Mohamed Gouda, Yasair S. S. Al-Fayiz

**Affiliations:** Department of Chemistry, College of Science, King Faisal University, Al-Ahsa 31982, Saudi Arabia

**Keywords:** functionalized-multi-walled carbon nanotubes, heavy metal ions removal, environment, Cd^2+^ and Pb^2+^ ions removal, adsorption, kinetic, isotherm, thermodynamic

## Abstract

In this work, acid-functionalized multiwalled carbon (MWCNTs–CO_2_H) nanotube was successfully functionalized with a heterocyclic scaffold, namely benzimidazole, to give novel functionalized multiwalled carbon nanotubes (BI@MWCNTs). Then, FTIR, XRD, TEM, EDX, Raman spectroscopy, DLS, and BET analyses were used to characterize the synthesized BI@MWCNTs. The effectiveness of the adsorption of two heavy metal ions, Cd^2+^ and Pb^2+^, in single metal and mixed metal solutions on the prepared material was investigated. Influencing parameters for the adsorption method, for example duration, pH, starting metal concentration, and BI@MWCNT dosage, were examined for both metal ions. Moreover, adsorption equilibrium isotherms fit with the Langmuir and Freundlich models perfectly, while the intra-particle diffusion models provide pseudo-second order adsorption kinetics. The adsorption of Cd^2+^ and Pb^2+^ ions onto BI@MWCNTs revealed an endothermic and a spontaneous method with great affinity as a result of the negative values of Gibbs free energy (ΔG) and the positive values of enthalpy (ΔH) and entropy (ΔS). Both Pb^2+^ and Cd^2+^ ions were completely eliminated from aqueous solution (100 and 98%, respectively) using the prepared material. Additionally, BI@MWCNTs have a high adsorption capacity and were regenerated in a simple way and reused for six cycles, which make them a cost-effective and efficient absorbent for the removal of such heavy metal ions from wastewater.

## 1. Introduction

According to Food and Agriculture Organization (FAO) of the United Nations report in 2020, “3.2 billion people live in agricultural areas with high to very high water shortages or scarcity, of whom 1.2 billion people—roughly one-sixth of the world’s population live in severely water-constrained agricultural areas”. The scarcity of water, mainly in arid and semi-arid areas of the world, is exerting exceptional pressure on sources and necessitates offering satisfactory water for human and different uses. Water recycle/reuse has been confirmed to be successful and promising for reliable water delivery. In many developing countries, this has led to the reuse of community wastewater in agriculture for irrigation. Nevertheless, it will increase human exposure to the pollutants. Inorganic pollutants form a major part of this problem, such as heavy metal ions. There are abundant metal ions which are significantly toxic to human beings and ecological environments, including lead (Pb), cadmium (Cd), mercury (Hg), zinc (Zn), nickel (Ni), etc. They exist, in various forms, in the soil and in natural and wastewater, and may become contaminants in food and drinking water. It was reported that plants grown and irrigated with untreated wastewater were highly polluted by heavy metals, which led to many health problems [1]. This was explained as a first result of heavy metal accumulation from irrigation water in soil [2]. Therefore, eliminating or decreasing the heavy metal contaminants in wastewater to safe limits is an important way to avoid their accumulation into the soil and, consequently, in the food chain. Various methods and technologies have been reported to achieve this goal, such as chemical precipitation and membrane separation, ion exchange, adsorption, [3,4,5,6,7], and nano-sized metal oxides [4], as well as carbon materials, such as carbon foam, carbon nanotubes, graphene and activated carbon [8,9,10,11], chitosan composites [12,13], and zeolites and weathered coal [14,15,16]. Precipitation and coagulation techniques have many drawbacks, such as producing large amounts of sludge [17]. Ion exchange is an expensive technology and requires pretreatment for wastewater to enhance the quality of the produced water [18,19]. Instead of offering high efficiency in the removal of heavy metals from highly concentrated aqueous solutions [20], membrane distillation (MD) technology suffers from some limitations, such as membrane fouling, low durability, high equipment and operating cost, and low permeation flux [21]. Adsorption has been reported to be the most commonly applied technique for the removal of heavy metal ions from water and wastewater. It is considered a flexible, convenient, and low-cost technology with a wide range of adsorbents that, after simple a desorption process, can be regenerated for multiple uses with high efficiency in many cases [4,8,22,23]. Given the increase in the worldwide manufacturing rate of CNTs, humans and ecosystems may be exposed to them (CNTs). Human health may be impacted, and environmental danger is increased. The lung toxicity brought on by MWCNTs inhalation has already been documented. Arul P. F. et al. have stated that a substance with anti-inflammatory and antioxidant properties may decrease the harmful impact brought on by CNTs by modification of MWCNTs with nano bis-dimethoxy curcumin (NBDMCA). They saw that NBDMCA might minimize the danger of inhalation toxicity brought on by MWCNTs [24,25].

Therefore, it is considered the most preferred process for decreasing the percentage of wastewater heavy metals pollution to safe and ecofriendly limits. In spite of numerous international efforts made over the last decades to bring solutions to solve this environmental problem, there are still important gaps to fill in. However, developing a specific sorbent with high adsorption capacity is still challenging task.

Herein we report a novel material based on heterocyclic moiety, namely 2-aminobenzimidazole. It was anchored and bonded directly to MWCNTs–CO_2_H via the carbonyl group using facile techniques. The prepared material was tested and different factors affecting the adsorption of dangerous metal ions, such as Cd^2+^ and Pb^2+^, from contaminated water solutions were investigated. Additionally, a range of adsorption experiments was used to study the adsorption kinetics, isotherms, thermodynamics, and removal capability of the synthesized materials.

## 2. Materials and Methods

### 2.1. Materials

The fine chemicals used in the experiments were purchased from Sigma-Aldrich Chemie GmbH, (Taufkirchen, Germany). All solvents used in experiments were HPLC grade and used as received without further purification. MWCNTs–CO_2_H was purchased from Chengdu Organic Chemicals Co, Ltd., (Chines Academy of Sciences, Chengdu, China). The MWCNTs had the following technical details: graphitized F-MWCNTs–COOH, -carboxylic group content: 0.25 wt.%, OD: >50 nm, length: 10–20 um, purity: 99.9%. Ammonium hydroxide (NH_4_OH), Cd(NO_3_)_2_.4H_2_O, and Pb(NO_3_)_2_ were purchased from Sigma Aldrich. All solutions were prepared using deionized water.

### 2.2. Preparation of the Functionalized MWCNT Materials

**Scheme 1:**
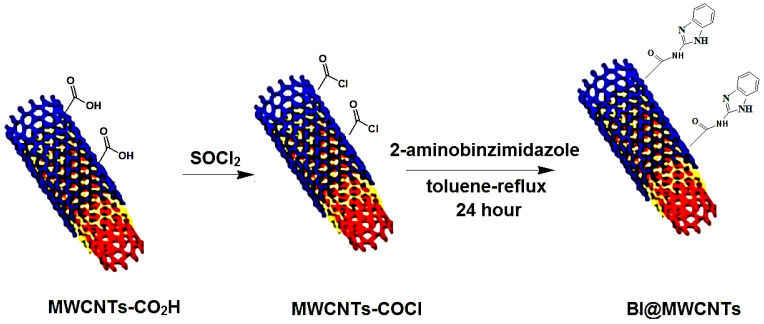
Chemically modified CO_2_H-functionalized MWCNTs with benzimidazole.

MWCNTs–COOH was treated with excess thienyl chloride to convert the carboxylic group into an acid chloride group (MWCNTs–COCl). The synthesized acid chloride was reacted with 2-aminobezimidazle directly to produce the functionalized MWCNTs with benzimidazole (BI@MWCNTs). The synthesis steps are explained hereafter. Firstly, the MWCNT-COOH (0.1 g) was sonicated with an excess of thienyl chloride for 2 h, then refluxed in a water bath for 24 h. The excess thienyl chloride was removed under vacuum (caution) to yield the acid chloride MWCNTs–COCl (~0.1 g). The obtained acid chloride was suspended in dry toluene (50 mL), and 2-aminobenzimidazole (0.1 g, 0.7 mmol) was added. The reaction mixture was subjected to sonication for 1 h, then reflux for 24 h. The hot reaction mixture was filtered off and washed with methanol. The resulting black solid was dried to yield BI@MWCNTs in an excellent yield (>95%, 0.1 g). The reaction steps are shown in Scheme 1.

### 2.3. Characterization of Functionalized MWCNT Materials

Data from an X-ray diffractometer (Shimadzu-7000, Torrance, CA, USA) using a CuK radiation beam (=0.154060 nm) was collected. Fourier transform infrared (FTIR) analysis with a range of 400 to 4000 cm^−1^ was used (A Bruker ALFA FTIR spectrometer, Billerica, MA, USA). Additionally, a scanning electron microscope (a JOEL scanning electron microscope-type electron probe (SEM) JXA-840, Tokyo, Japan) was used to investigate the surface morphology of the synthesized nanomaterials. An XploRA PLUS device (Horiba, Kyoto, Japan) verified the Raman spectra. Furthermore, particle size distribution of prepared samples was characterized using dynamic light scattering (DLS) that was carried out by using Otsuka Electronics DLS-6500 system with the He-Ne laser (λ = 633 nm, 10 mW). The temperature of the sample solution was controlled at ~25 °C. In addition, the surface area and total pore volume of Brunauer–Emmett–Teller (BET) structures were determined.

### 2.4. Adsorption Tests

Applying a thermostatic shaker set at 150 rpm, a specified weight of BI@MWCNTs was added to 50 mL of worked solutions of metal salt at 25 °C in order to conduct the adsorption assays. By diluting 1 g/L standard solution of Pb(NO_3_)_2_ or Cd(NO_3_)_2_.4H_2_O with deionized water, altered starting metal solution concentrations (50, 100, 150, and 200 mg/L) were created. Subsequently, for the adsorption operations, the liquid and solid phases of the BI@MWCNTs were separated using centrifugation at 4000 rpm for 15 min using atomic adsorption spectroscopy; the amount of remaining metal in the solution was calculated. The quantities of adsorbed metal ions were calculated using the following equations:

(1) $$q_{t}=\frac{\left(C_{0}-C_{t}\right)}{m}\cdot V$$

(2) $$q_{e}=\frac{\left(C_{0}-C_{e}\right)}{m}\cdot V$$

where the quantities of adsorbed metal per unit weight of the adsorbent at time (t) and at equilibrium are represented by the variables (q_t_) and (q_e_) (mg g^−1^), respectively. The metal concentrations are (C_0_, C_t_, and C_e_) (mg L^−1^) at the beginning time, time t, and at equilibrium time, respectively. V (L) denotes the volume of the metal solution, and m (g) is the adsorbent’s weight. The percent of removal (R%) was determined as follows:

(3) $$R\%=\frac{\left(C_{0}-C_{t}\right)}{C_{0}}\times 100$$

The following equation was used to evaluate the distribution coefficient (K_d_) of Cd^2+^ and Pb^2+^ onto the produced adsorbent:

K_d_ = q_e_/C_e_ (4)

When evaluating the actual performance of the adsorbent, the distribution coefficient or partition coefficient (K_d_) may be utilized.

The following equation was used to investigate the selectivity coefficient (α) for the obligatory nature of a certain adsorbate in the presence of intrusive ions:

α = K_d_ (T)/K_d_ (I) (5)

where K_d_ (I) is the K_d_ value of the other metal in the multi-metal solutions, which in this case is cadmium, and K_d_ (T) is the K_d_ value of the targeted metal.

### 2.5. Adsorption Kinetics Process

Since they represent the rate at which an adsorbent is absorbed, kinetic studies are crucial. Based on kinetic studies, the rate of adsorption method and the mechanism of the adsorption can be clarified. Based on kinetic measurements with starting Cd^2+^ and Pb^2+^ ion concentrations ranging from 50 to 200 mg/L, the pH was set at 5.5 (to prevent precipitate of hydroxyl) and the adsorption duration was varied from 1 to 30 min with a dosage of 0.02 mg/mL of adsorbent for our kinetic investigations. The pseudo-first order and pseudo-second order models were used to clarify the kinetics of adsorption [26,27]. Equations (6) and (7) are as follows:

q_t_ = q_e_ [1 − exp(−k_1_t)] (6)

(7) $$q_{t}=\frac{k_{2}q_{e}2t}{1+k_{2}q_{e}t}\times 100$$

where k_1_ and k_2_ are the adsorption rate constants of the pseudo-first- and pseudo-second order, respectively.

For low solute concentrations, the pseudo-first order kinetic model is more appropriate. One way to express it in writing is explained as follows:

ln (q_e_ − q_t_) = ln q_e_ − k_1_t (8)

where k_1_ (min^−1^) is the rate constant of the pseudo-first order adsorption, q_e_ is the amount of metal adsorbed at saturation per gram of adsorbent, and q_t_ is the amount of metal adsorbed at time t per gram of adsorbent (mg gm^−1^), respectively. The pseudo-second order kinetic model depends on both the quantity of solute adsorbed at equilibrium and the amount adsorbed on the surface of the adsorbent.

An intra-particle diffusion model was applied at various starting concentrations to examine the diffusion mechanism of Cd(II) and Pb(II) onto produced BI@MWCNT adsorbents [27], as follows:

q_t_ = k_i_ t^½^ + C (9)

where C is the magnitude of the intercept, a constant that indicates the importance of the boundary layer or mass transfer effect, and k_i_ (mg g^−1^ min^−1/2^) is the intra-particle diffusion rate constant, which is the slope of the straight line of q_t_ vs. t^1/2^.

### 2.6. Adsorption Isotherms

Using the Langmuir and Freundlich isotherms, the adsorption thermodynamics and isotherms were put to the test to confirm the metal uptake behavior of the BI@MWCNTs. The adsorption isotherms was used to show the sorption capacity of the BI@MWCNTs at various starting concentrations at equilibrium. Adsorption isotherms provide a full understanding of the nature of interactions by describing how the adsorbate interacts with adsorbents. For this investigation, a number of isotherm equations have been generated and used, and the two significant isotherms were utilized. By utilizing several adsorption isotherm models, the mechanism of interaction between the adsorbed metal and BI@MWCNTs was investigated. The Langmuir and Freundlich models suit the experimental data well [28,29]. The Langmuir model is stated as follows and takes into account the progression of an adsorbate monolayer on an adsorbent’s normal surface:

(10) $$\frac{C_{e}}{q_{e}}=\frac{1}{k_{L}}+\frac{C_{e}}{q_{m}}$$

where C_e_ is the equilibrium adsorbate concentration (in mmol L^−1^), q_e_ is the quantity of adsorbate adsorbed per unit weight of adsorbent (in mmol g^−1^), q_m_ is the adsorption capacity (mmol g^−1^), or monolayer capacity, and k_L_ is a constant (L mmol^−1^), as shown in the following equations [9,28]. A key feature of the Langmuir model is the separation factor (R_L_), a dimensionless quantity. The following is how the R_L_ equation is written out:

(11) $$R_{L}=\frac{1}{\left(1+k_{L\;}C_{o}\right)}$$

where k_L_ is the Langmuir constant (L mg^−1^) and C_o_ is the adsorbate’s maximum initial concentration (mg L^−1^). The value of R_L_ determines whether the isotherm’s form is irreversible (R_L_ = 0), favorable (0 < R_L_ < 1), linear (R_L_ = 1), or unfavorable (R_L_ > 1). The Freundlich isotherm, on the other hand, takes into account adsorption between adsorbates and adsorbents with a heterogeneous surface. The energy level at the adsorption sites reveals that the rate of adsorption varies. In order to obtain Freundlich isotherms, we use the following equation:

(12) $$\ln q_{e}=\ln k_{f}+\frac{1}{\left(n\ln C_{e}\right)}$$

where k_f_ is a constant (function of temperature and adsorption energy) and n is a constant related to adsorption intensity, determined by plotting ln q_e_ vs. ln C_e_, which gives a straight line with slope of 1/n and an intercept of ln k_f_. The magnitude of the “n” shows an indication of the favorability of adsorption.

### 2.7. Adsorption Thermodynamics

At 25 °C and 35 °C, thermodynamic adsorption studies were conducted with an initial concentration of 100 mg L^−1^. The following equations [27,30] were used to compute the thermodynamic parameters:

K_D_ = q_e_/C_e_ (13)

ΔG = −R T lnK_D_ (14)

lnK_D_ = (ΔS/R) − (ΔH/RT) (15)

where K_D_ is the equilibrium partition constant, ΔG is the Gibbs free energy change (kJ mol^−1^), R is the universal gas constant (8.314 J mol^−1^ K^−1^), T (K) is the temperature, ΔH is the enthalpy change (kJ mol^−1^), and ΔS is the entropy change (kJ mol^−1^ K^−1^).

The K_D_ values for each temperature were used to calculate the values of ΔG, while the slope and intercept of the ln K_D_ vs. 1/T plot were used to determine the values of ΔH and ΔS, respectively.

### 2.8. Test for Regeneration and Reuse

Evaluation of an adsorbent’s practical viability requires regeneration and reuse tests. In order to test the reversibility of metal ion adsorption with BI@MWCNTs, 4 mg of BI@MWCNTs were combined with 100 mL of 50 mg L^−1^ Cd(II) and Pb(II). The mixtures were kept for 15 min with shaking at 120 rpm. The adsorbent was separated from the mixture by centrifugation. Separated adsorbents were washed, dried in an oven at 40 °C for two hours, and then reused. A total of 0.1 M HCl was added after the adsorption process, and the solution was then ultrasonically treated at ambient temperature for 30 min. Metal ion concentration was determined using atomic adsorption spectroscopy. To investigate regenerability, three cyclic adsorption–desorption processes were used.

## 3. Results and Discussion

### 3.1. Characterization of BI@MWCNTs

#### 3.1.1. FTIR

**Figure 1:**
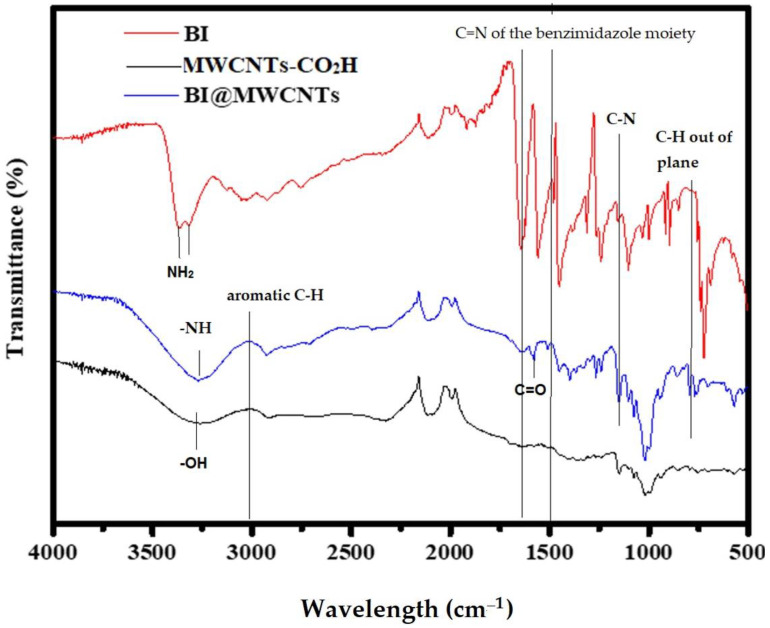
FTIR spectra of BI, MWCNTs–CO_2_H, and BI@MWCNTs.

To investigate the successful functionalization of the MWCNTs–CO_2_H with BI, the infrared spectra of BI, MWCNTs–CO_2_H, and BI@MWCNTs were verified. Figure 1 shows the FTIR spectra of BI, MWCNTs–CO_2_H, and BI@MWCNTs. The spectra indicate the disappearance of the primary amino pair peak of the aminobenzimidazole in the prepared BI@MWCNTs with the presence of the -OH absorption band of the remaining unfunctionalized carboxylic group at 3333 cm^−1^. A clear peak, which could be attributed to conjugated C=O stretching vibrations, was seen at 1630 cm^−1^. The presence of additional peaks in the range of 1600–1500 cm^−1^ were attributed to the aromatic ring system and C=N of the benzimidazole moiety. Furthermore, the 3020 cm^−1^, 1215 cm^−1^, and 769 cm^−1^ peaks in the BI@MWCNTs’ spectrum, which are brought on by the aromatic C-H stretching, C-N stretching, and C-H out of plane bending modes, respectively, confirmed the functionalization of MWNCTs-CO_2_H with BI [31,32].

#### 3.1.2. SEM

**Figure 2:**
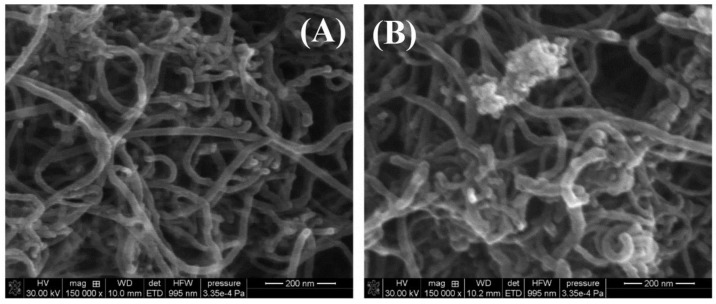
SEM image of (**A**), MWCNTs–CO_2_H and (**B**), BI@MWCNTs.

Figure 2 displays the MWCNTs–CO_2_H and BI@MWCNTs images from the scanning electron microscopy. The MWCNTs–CO_2_H, which have a diameter of about 14 nm, are shown in Figure 2A. The newly prepared material, BI@MWCNTs, is shown in Figure 2B. The BI adsorption causes the nanotubes’ diameter to grow up to 20 nm and gives them their distinctive shading.

#### 3.1.3. Raman Spectroscopy

**Figure 3:**
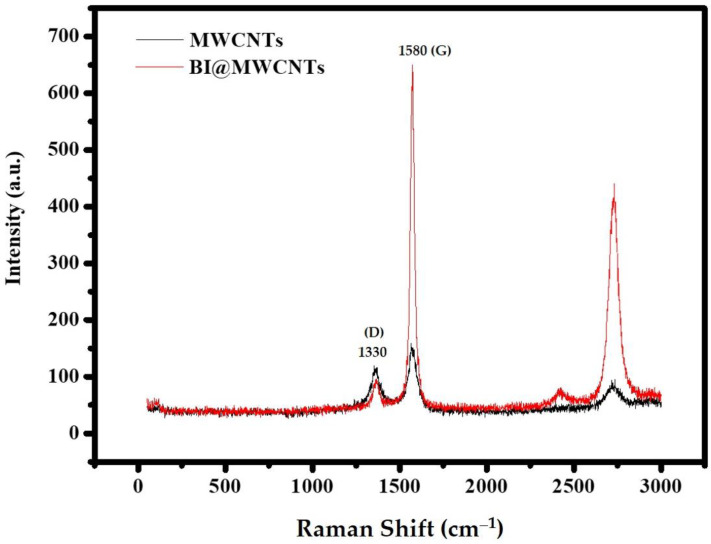
Raman spectra of MWCNTs–CO_2_H and BI@MWCNTs.

Raman spectra provided additional support for MWCNTs functionalization. As shown in Figure 3, both the spectra for MWCNTs–COOH and BI@MWCNTs contain the distinctive G (tangential mode) and D (disorder mode) bands linked to the sp^2^ carbon and the carbon nanotube at 1585 cm^−1^ and 1330 cm^−1^, respectively. Furthermore, the intensity of the D band for the MWCNTs–CO_2_H is more than the intensity of the D band for the BI@MWCNTs, which confirmed that the functionalization with BI had successfully occurred. In addition, the intensity ratio ID/IG for BI@MWCNTs (0.98) was higher than that for MWCNTs–CO_2_H (0.85), which provided additional proof that the out of the plane rotation of the heterocyclic side chain contributed more to these materials than MWCNTs–CO_2_H [32].

#### 3.1.4. XRD

**Figure 4:**
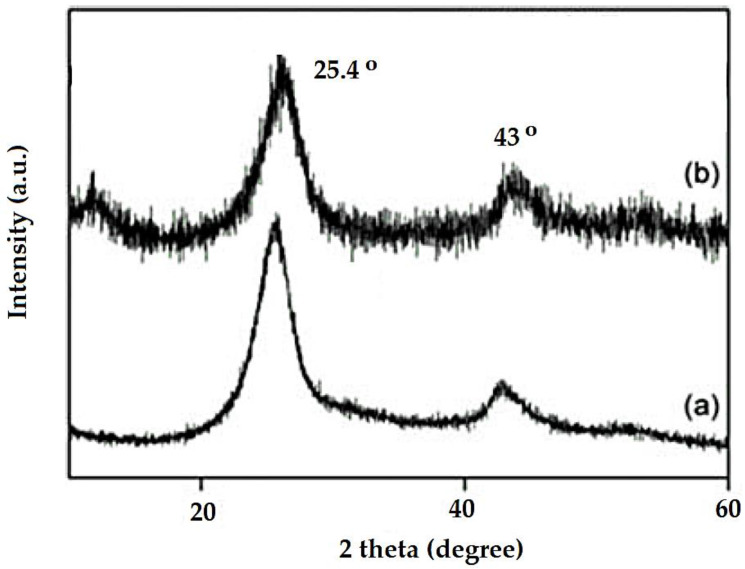
XRD pattern of (a), MWCNTs–CO_2_H and (b), BI@MWCNTs.

The recorded MWCNTs–CO_2_H and BI@MWCNTs X-ray diffraction (XRD) patterns are shown in Figure 4 as intensity counts vs. 2θ. One strong diffraction peak at 2θ = 25.4°, which corresponds to the (002) plane, and one low-intensity peak at 2θ = 43°, which corresponds to the (100) reflection plane, were identified in the MWCNTs–CO_2_H and BI@MWCNTs’ diffractograms (JCPDS card no. 75-1621) and were attributed to the graphitic structure. When MWCNTs–CO_2_H was converted to BI@MWCNTs, no appreciable changes in the 2θ values of the XRD patterns were seen. The enhanced crystallinity of BI@MWCNTs is evidenced by the fact that the diffraction peak intensity of BI@MWCNTs is higher than that of MWCNTs–CO_2_H. This might be because when MWCNTs–CO_2_H underwent treatment, reaction process led to more purification and the removal of amorphous carbon and other impurities in the carbon nanotube.

#### 3.1.5. Dynamic Light Scattering (DLS)

**Figure 5:**
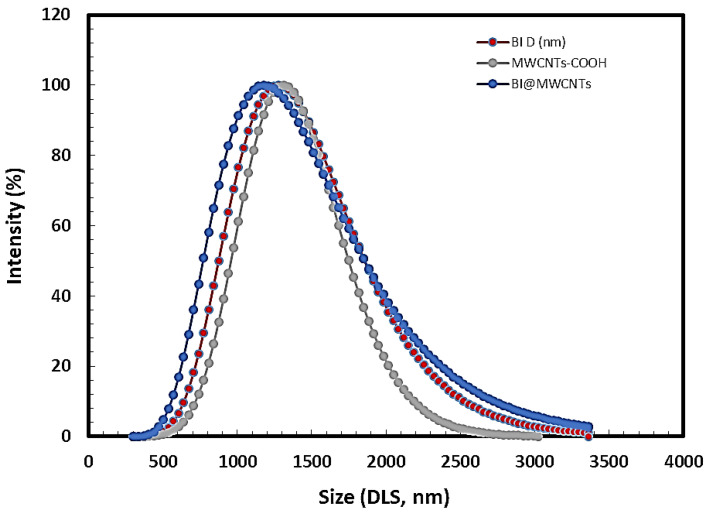
Size distribution by intensity obtained with DLS.

Dynamic light scattering (DLS) analyses of 2-aminobenzimidazole (BI), MWCNTs–CO_2_H, and the functionalized carbon nanotubes with benzimidazole (BI@MWCNTs) were studied to affirm the particle size distribution. The samples were analyzed throughout the experimental period, from 0 to 3 h, to study the dispersion stability of the material in glyceline and water, as a reference. All samples were dispersed in glyceline and water to suitable concentrations and were analyzed in triplicate. The obtained size of the prepared particles is bigger than what was previously observed through SEM and ranged from 600 to 2000 nm, because the DLS investigation measures the hydrodynamic diameter and the characteristics of nanoparticles exclusively depending on their size. There are numerous techniques available for describing the surfaces of nanoparticles. Due to the differences in how to measure the particle dimensions, the pore size distribution values of functionalized MWCNTs are different from those of SEM (Figure 2) compared to DSL, as shown in Figure 5. This is due to the fact that particles are suspended in a solution for particle size distribution (DLS), and measurements are taken while the particles are in solution using Brownian motion [33]. The hydrodynamic diameter may increase as a result of the individual particles aggregating.

#### 3.1.6. BET Analysis

**Figure 6:**
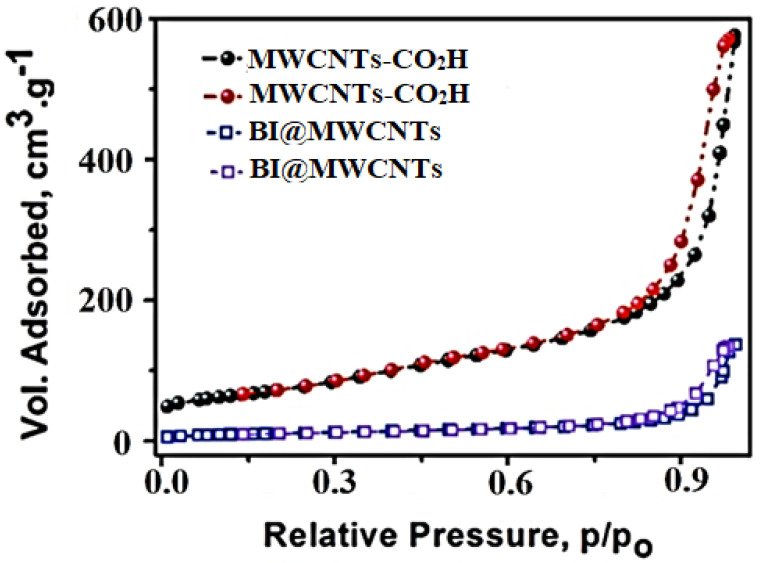
N_2_ adsorption–desorption isotherms of MWCNTs–CO_2_H and BI@MWCNTs.

Brunauer–Emmett–Teller (BET) analysis was used to measure the specific surface areas of the adsorbents, and an automatic surface analyzer was used to measure the total pore volume and pore size (Quantachrome, Boynton Beach, FL, USA). Figure 6 shows N_2_ adsorption isotherms, which measured at 77 K, and nitrogen adsorption–desorption isotherms for the MWCNTs–CO_2_H and BI@MWCNTs. The result is summarized in Table 1 with the BET measurements of the MWCNTs–CO_2_H and BI@MWCNTs. Due to the intertubular structure, hysteresis was seen for both MWCNTs–CO_2_H and BI@MWCNTs. The noticed decrease effect may be attributed to the well-known capillary condensation [16]. The isotherms obtained from the various samples were categorized as type IV isotherms with H3 type hysteresis loops by the original IUPAC classification [16]. This finding suggests that samples may contain mesoporous and macroporous structures. The microporous structure with a mean pore size of 14 nm in the sample is responsible for the abrupt increase in the N_2_ adsorption plot of MWCNTs–CO_2_H at a relative pressure of > 0.9. Furthermore, the MWCNTs–CO_2_H have high surface area (252.6 m^2^ g^−1^) and high pore volume (0.799 cm^3^ g^−1^), implying the presence of additional sites, functionalization with BI, and co-lateral growth of BI. In contrast, the BI@MWCNT surface area and pore volume were decreased to 45.3 m^2^ g^−1^ and 0.3041 cm^3^ g^−1^, respectively.

### 3.2. Adsorption Behaviors

#### 3.2.1. Effect of pH

**Figure 7:**
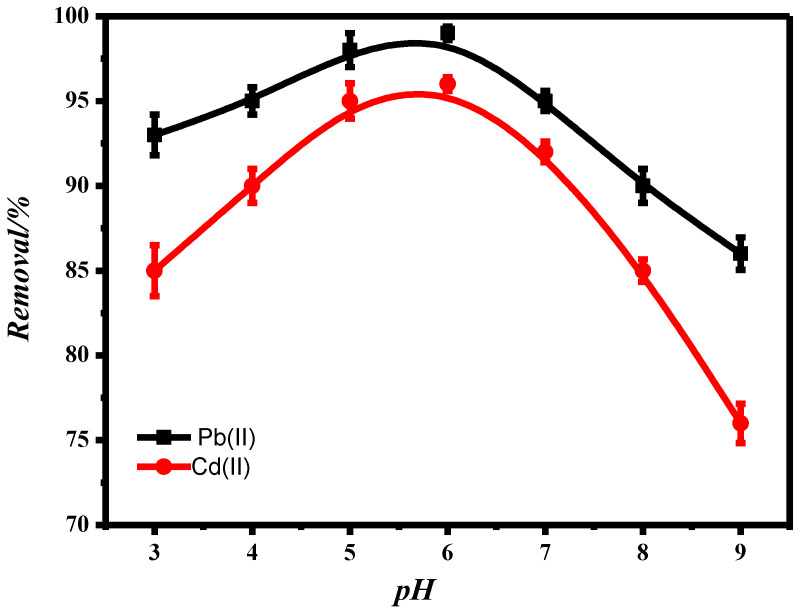
Removal percentage of Pb^2+^ and Cd^2+^ adsorbed onto BI@MWCNTs at different pH solutions of metal salt solution (C_0_: 100 mg/L; dosage of adsorbent: 0.2 mg/mL; temperature: 25 °C; time: 30 min; volume: 50 mL).

One of the key influences regulating the method of the adsorption is the solution pH, which has an impact on how the adsorbate interacts with the adsorbent surface charges. In order to find the ideal pH level, the influence of the pH on the adsorption effectiveness of BI@MWCNTs toward Pb^2+^ and Cd^2+^ was investigated in the pH range of 2.5 to 10. The percentage of removal significantly enhanced after the pH was raised from 3 to 6, as shown in Figure 7. Nevertheless, the removal effectiveness progressed slowly when the solution was in the range of 5–6. The quantity of adsorption reduced after pH surpassed 7. The Pb^2+^ and Cd^2+^ adsorption behavior on the produced BI@MWCNTs in relation to pH values might be explained by a variety of factors. For instance, at lower pH values (less than 4), the surfaces of the BI@MWCNTs would be surrounded by a large number of H_3_O^+^ ions that contest with the metal ions. Furthermore, at lower pH values (less than 4), the nitrogen atoms of the benzimidazole scaffold (either the pyridine- or pyrrole-like) might be easily protonated, which would subsequently affect the adsorption efficiency of the BI@MWCNTs. However, increasing the pH of the solution to higher values (the weak acidic condition) to a range of 5–6 increases the electrostatic attraction between the metal ions and the surface of the BI@MWCNTs, progressively improving the removal effectiveness [34]. Furthermore, Pb^2+^ and Cd^2+^ are present in varied formulae in solution at altered pH levels, while when the pH exceeds 7, the precipitates of hydroxides (Cd(OH)_2_., Cd(OH)_3−_Pb(OH)_2_, and Pb(OH)_3_) are developed rapidly, and these precipitates are hardly adsorbed onto the BI@MWCNTs [34]. This is because the benzimidazole nucleus has high-class structural features and an electron-rich scaffold, which might produce an electrostatic repulsion. At a pH greater than 8, the adsorption of Pb^2+^ and Cd^2+^ fall significantly and it becomes problematic to eliminated using BI@MWCNTs. Therefore, in the next studies, a pH of 5 will be the pH of choice for eliminating Pb^2+^ and Cd^2+^ and will be applied in this study.

#### 3.2.2. Adsorption Competition

**Figure 8:**
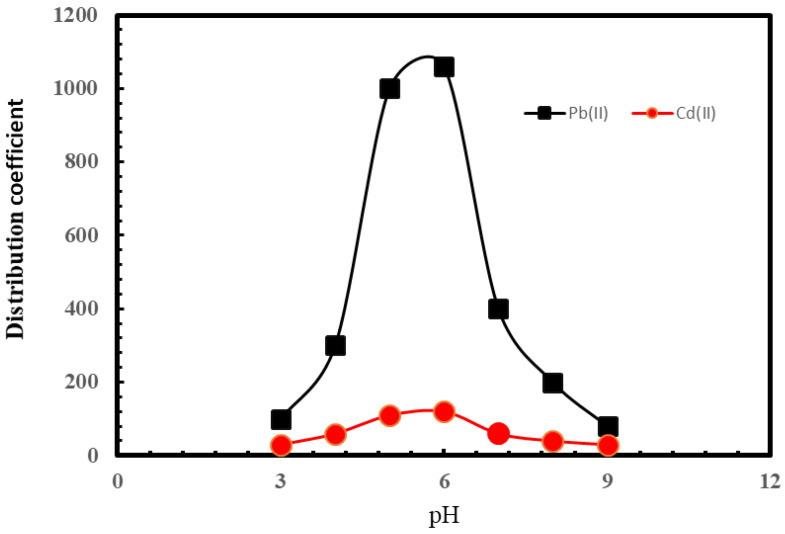
Distribution coefficient for Pb^2+^ and Cd^2+^ adsorbed onto BI@MWCNTs at different pH.

Affording to adsorbent’s adsorption capacity ratio to the final concentration of adsorbate in the liquid at equilibrium, the distribution coefficient (K_d_) essentially signifies the accurate presentation of an adsorbent [31,35], whereas the adsorption capacity and removal effectiveness can be changed by operational settings. In this investigation, Equation (5) was utilized to assess the effectiveness of the produced adsorbent material using the distribution coefficient (K_d_) for Pb^2+^ and Cd^2+^. The values of K_d_ for Pb^2+^ and Cd^2+^ increased with increasing pH to reach pH 6 and then decreased by further pH increasing, as shown in Figure 8. This is because higher pH activates more adsorption locations and makes extra metal ions accessible to transfer from the solution to the BI@MWCNTs’ surface. Additionally, at each pH level, BI@MWCNTs demonstrated a more definite favorite for Pb^2+^ over Cd^2+^. These can be attributed to Pb^2+^ showed strong affinity for BI@MWCNTs in the solution containing two metals. Table 2 shows the distribution coefficient values of Pb^2+^ are more than that of Cd^2+^, and this is showing that BI@MWCNTs exhibited selective adsorption for Pb^2+^ over Cd^2+^ according to distribution coefficient values onto BI@MWCNTs.

#### 3.2.3. Pb^2+^ and Cd^2+^ Adsorption Optimization

**Figure 9:**
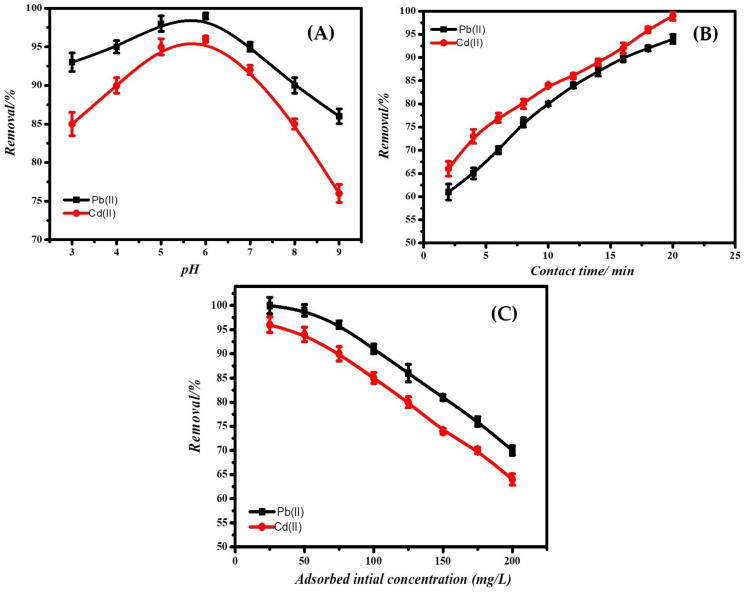
Removal% of Pb^2+^ and Cd^2+^ using (**A**), different contact time (min); (**B**), different initial adsorbed concentration (mg/L), and (**C**), different BI@MWCNTs dosage at pH 5 with three trials of each factor.

The optimization of the adsorption processes of Pb^2+^ and Cd^2+^ onto BI@MWCNTs, was accomplished by utilizing three factors, namely time (min), metal starting concentration (mg/L), and BI@MWCNT dosage (mg). The pH of the solutions was maintained at 5 during the whole process period. Figure 9 shows that the percentage of removal efficiency was increased with longer contact time before decreasing with initial metal concentrations from 50 to 150 mg/L. The percent removal then remained roughly constant. This observation showed that the metal ions were initially superficially adsorbed, and the rate of adsorption increased quickly. The metal ions began to adsorb into the porous structure of BI@MWCNTs and reached a constant value due to the external surface of the BI@MWCNTs becoming saturated, meaning that no further adsorption took place. The optimum values of the three parameters, as shown in Figure 9A–C, were 20 min contact time, 50 mg/L of Pb^2+^ and Cd^2+^ concentrations, and 6 mg of BI@MWCNTs at a pH of 5.0 and temperature of 25 °C in order to completely remove Pb^2+^. However, the optimum removal% of Pb^2+^ and Cd^2+^ was 100% and 98%, respectively, using 6 mg of BI@MWCNTs, pH 5.0, and an initial metal ion concentration 50 mg/L, and a contact time 20 min was accomplished. Using statistical analysis conditions, a confirmation test (three replicates) was conducted.

### 3.3. Adsorption Kinetics

**Figure 10:**
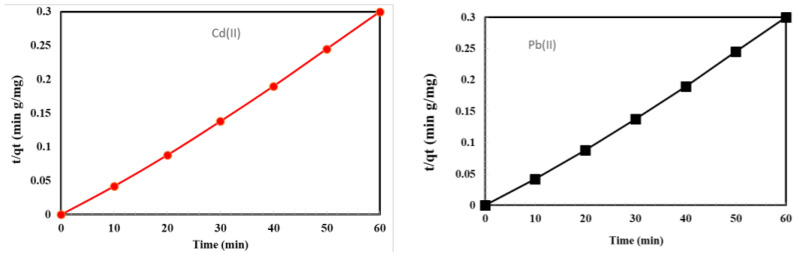
Pseudo-second order kinetic models for the adsorption of Pb^2+^ and Cd^2+^ on BI@MWCNTs (conditions: pH, 5.0; initial adsorbed concentration, 50 mg/L; adsorbent dose, 4 mg; temperature, 25 °C).

The influence of varying the adsorption time on the rate of the adsorption process was examined by applying two models of kinetics, namely the pseudo-first order and pseudo-second order models. These models were useful in understanding the Pb^2+^ and Cd^2+^ adsorption mechanisms using the prepared BI@MWCNTs at the adsorbed initial concentration (50 mg/L). The adsorption capacity was thought to be controlled by a single mechanism performing on a single class of adsorbing sites according to the pseudo-first order theory [28]. The calculated values were found to be very different from their similar experimental values when the calculated values were compared to the experimental adsorption capacities. Consequently, the pseudo-first order reaction kinetic model could not effectively characterize Pb^2+^ and Cd^2+^ adsorption on BI@MWCNTs. Alternatively, Figure 10 shows that the pseudo-second order kinetic model was reliant on the quantity of adsorbed adsorbate on the surface of the BI@MWCNTs. The agreement between the calculated (q_e,cal_) and experimental q_e_ values is shown in Table 3. As demonstrated in Table 3, the calculated q_e_ (q_e,cal_) values agreed with the experimental q_e_ values (q_e,exp_), and the correlation coefficient (R^2^) values that were attained were consistently higher than those of the pseudo-first order. According to the obtained results, chemisorption was the rate-determining step in the adsorption procedure, and the adsorption rate for both ions was influenced by the approachability of adsorption locations on the surface of the BI@MWCNTs. Furthermore, *k*_2_ values decrease with the increase in the initial concentrations of Pb^2+^ or Cd^2+^, which may be due to higher competition for the adsorption sites at high concentrations compared with low concentrations [36].

### 3.4. Adsorption Isotherms

As shown in Table 4, using the Langmuir and Freundlich adsorption isotherm models at dissimilar initial concentrations and two altered temperatures (25 °C and 35 °C), the data revealed that the Langmuir model, which suggests that Pb^2+^ and Cd^2+^ ions adsorb with monolayer coverage, best represented the adsorption data. The determined value q_m_ of Cd^2+^ adsorbed on the BI@MWCNTs was 916 mg/g, whereas the determined value q_m_ of Pb^2+^ adsorbed on the BI@MWCNTs was 931 mg/g, which was noticed to be increased at high temperatures. In addition, the Freundlich model’s 1/n_F_ values, which indicate surface heterogeneity or adsorption intensity, varied between 0 and 1, revealing a larger heterogeneity as the values move toward 0. The Pb^2+^ and Cd^2+^ ions adsorbed onto the BI@MWCNTs primarily via the internal surface by regulatory intra-particle diffusion, which is consistent with the conclusion that a heterogeneous chemisorption process took place. The findings show that both metals can effectively adsorb using the Langmuir and Freundlich isotherm models.

### 3.5. Adsorption Thermodynamics

Table 5 shows the thermodynamic factors that were investigated at 25 °C, including Gibbs free energy (ΔG), enthalpy (ΔH), and entropy (ΔS). The data show that the negative values of ΔG indicate that the adsorption system on the BI@MWCNTs was physisorption and a spontaneous process [37]. On the other hand, the positive values of Δ*H* and Δ*S* elucidate that the adsorption processes on the BI@MWCNTs were random and endothermic processes, respectively [27].

### 3.6. Regeneration Adsorption Tests

**Figure 11:**
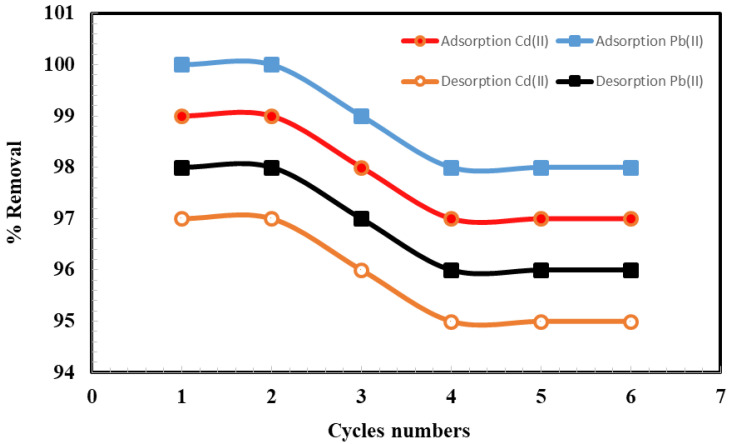
Pb^2+^ and Cd^2+^ adsorption-desorption cycles on BI@MWCNTs (adsorbed initial concentration, 50 mg/L; adsorbent dose, 4 mg/L; pH, 5.0; temperature, 25 °C).

After undergoing the adsorption process, 4 mg of the used BI@MWCNTs was spread in 0.1 M HCl solution and sonicated for 30 min with the intention of assessing its ability to be used again. The BI@MWCNTs were then cleaned and reused. Figure 11 shows the percentage of removal as a motionless high after five cycles, and the differences were relatively minor. This is a strong indication that BI@MWCNTs are an excellent and cost-effective adsorbent for both lead and cadmium elimination in wastewater applications.

### 3.7. Comparative Study

Table 6 shows comparative values of the previously reported percentages of removal for Pb^2+^ and Cd^2+^ from solutions using different carbon nanotube-, carbon-, and graphene oxide-based materials with the prepared BI@MWCNTs in this study. As illustrated, the percentage of removal, using the synthesized BI@MWCNTs in this study for (Pb^2+^) and (Cd^2+^) are 100 and 98%, respectively, which are excellent values for the elimination of both metal cations compared to the previously reported carbon-based adsorbents. Furthermore, the prepared functionalized MWCNTs in this study (BI@MWCNTs) have been recovered and reused six times, which makes them a cost-effective and efficient absorbent for the removal of (Pb^2+^) and (Cd^2+^) from wastewater.

## 4. Conclusions

A well-known functionalized benzimidazole was used to modify acid functionalized multiwalled-carbon nanotubes (MWCNTs–CO_2_H) and yield a novel functionalized nanomaterial (BI@MWCNTs). The structure and physical characteristics of the BI@MWCNTs were confirmed and measured by surface area analysis, dynamic light scattering (DLS) analysis, Raman spectroscopy, scanning electron microscopy (SEM), and FT-IR. The main conclusions of this study can be discussed in the following points: Effect of pH—the influence of the pH on the adsorption effectiveness of BI@MWCNTs toward Pb^2+^ and Cd^2+^ was investigated in the pH range of 2.5 to 10. The optimum efficiency for removal of both ions was achieved at pH 5. BI@MWCNTs exhibited selective adsorption for Pb^2+^ over Cd^2+^ according to distribution coefficient values. The optimization of the adsorption processes of Pb^2+^ and Cd^2+^ onto BI@MWCNTs was accomplished by utilizing three factors, namely time (min), metal starting concentration (mg/L), and BI@MWCNT dosage (mg). The optimum values of the three parameters are 20 min contact time, 50 mg/L of Pb^2+^, and Cd^2+^ concentrations, and 6 mg of BI@MWCNTs, carried out at a pH of 5.0 and a temperature of 25 °C in order to completely remove Pb^2+^ (100%) and 98% of Cd^2+^. In terms of adsorption kinetics, the influence of varying the adsorption time on the rate of the adsorption process was examined. According to the obtained results, chemisorption was the rate determining step in the adsorption procedure, and the adsorption rate for both ions was influenced by the approachability of adsorption locations on the surface of the BI@MWCNTs. The pseudo-second order kinetic model was reliant on the quantity of adsorbed adsorbate on the surface of the BI@MWCNTs. In terms of adsorption isotherms, using the Langmuir and Freundlich adsorption isotherm models at dissimilar initial concentrations and two altered temperatures (25 °C and 35 °C), the data revealed that the Langmuir model, which suggests that Pb^2+^ and Cd^2+^ ions adsorb with monolayer coverage on the BI@MWCNTs’ surface, best represented the adsorption data. The thermodynamic factors that were investigated at 25 °C, including Gibbs free energy (ΔG), enthalpy (ΔH), and entropy (ΔS), gave the following results. The Pb^2+^ and Cd^2+^ adsorbent systems were shown to be spontaneous by the negative values of ΔG, while the positive values of ΔS show that randomness increases at the solid–liquid interface during the adsorption process, and the positive values of ΔH show that the adsorption route is endothermic. Regeneration adsorption tests have revealed that the percentage of removal as a motionless high after five cycles, and the differences were relatively minor. The comparative study revealed that the percentage of removal using the prepared BI@MWCNTs for (Pb^2+^) and (Cd^2+^) are 100 and 98%, respectively, which are excellent values for the elimination of both metal cations compared to the previously reported carbon-based adsorbents. Furthermore, the prepared functionalized MWCNTs in this study (BI@MWCNTs) have been recovered and reused six times, which make them a cost-effective and efficient absorbent for the removal of (Pb^2+^) and (Cd^2+^) from wastewater.

## Figures and Tables

**Table 1:** Surface area of MWCNTs–CO_2_H and BI@MWCNTs.

Type	BET Surface Area (m^2^/g)	Pore Size (nm)	Pore Volume (m^3^/g)
MWCNTs–CO_2_H	252.6	14	0.7999
BI@MWCNTs	45.3	20	0.3041

**Table 2:** Adsorption presentation of BI@MWCNTs applied for the removal of Pb^2+^ and Cd^2+^ at a pH 5 and 100 mg/L metal start concentration.

	Maximum Adsorption Capacity (mg/g)		Maximum (%) Removal		Distribution Coefficient (L/g)	
	Pb^2+^	Cd^2+^	Pb^2+^	Cd^2+^	Pb^2+^	Cd^2+^
BI@MWCNTs	485	470	97	94	1000	110

**Table 3:** The kinetic model parameters and determination coefficients for Pb^2+^ and Cd^2+^ adsorption on prepared BI@MWCNTs.

	Cd(II) Initial Concentrations (mg/L) Adsorbed on BI@MWCNTs			Pb(II) Initial Concentrations (mg/L) Adsorbed on BI@MWCNTs		
	50	150	200	50	150	200
q_e,exp_ (mg/g) ^a^	350	490	970	380	500	1017
Pseudo-first order						
q_e,cal_ (mg/g)	32	57	850	0.36	4.2	35
k_1_ (min^−1^)	0.16	0.17	0.21	0.05	0.08	0.1
R^2^	0.7	0.88	0.96	0.92	0.89	0.80
Pseudo-second order						
q_e,cal_ (mg/g)	356	499	990	380	500	1016
K_2_ (min^−1^)	0.003	0.0005	0.0006	0.98	0.07	0.01
R^2^	0.999	1	1	0.999	1	1

^a^ The equilibrium time for the removal of both cadmium and lead was 20 min.

**Table 4:** The parameters of the adsorption isotherm for the adsorption of Pb^2+^ and Cd^2+^ on BI@MWCNTs using the dose of the adsorbent at 4 mg/mL.

	Cd(II) Adsorption on BI@MWCNTs		Pb(II) Adsorption on BI@MWCNTs	
	25 °C	30 °C	25 °C	30 °C
Langmuir isotherm				
q_m_ (mg/g)	840	916	930	931
K_L_ (L/mg)	0.45	0.73	0.8	0.81
R^2^	0.997	0.998	0.999	0.999
R_L_	0.008	0.008	0.0069	0.0069
Freundlich isotherm				
K_F_ (mg/g)	220	216	228	232
1/n_F_	0.22	0.21	0.24	0.21
R^2^	0.998	0.998	0.997	0.995

**Table 5:** Thermodynamic conditions for 50 mg/L Pb^2+^ and Cd^2+^ adsorption on BI@MWCNTs at 25 °C.

T (K)	ΔS (J mol^−1^ K^−1^)		ΔG (kJ mol^−1^)		ΔH (kJ mol^−1^)	
	Cd(II)	Pb(II)	Cd(II)	Pb(II)	Cd(II)	Pb(II)
298	40.38760	53.2325	−10,971	−16,325.7	2.97768	2.91339

**Table 6:** Comparison between BI@MWCNTs and previously reported carbon-based adsorbents in terms of effectiveness at removing Pb^2+^ and Cd^2+^ from water.

Carbon-Based Materials	% Removal		Reference
	Pb^2+^	Cd^2+^	
Graphene oxide (GO)/isophorone diisocyanate	70	40	[38]
Ceramic supported GO/attapulgite composite	99	77	[39]
GO framework/ethylenediamine	89	80	[40]
MWCNTs–hydroxyquinoline	95	89	[41]
MWCNT–CO_2_H	88	-	[22]
MoS_2_/SH–MWCNTs	95	80	[42]
Ag-coated SWCNTs	93	15	[43]
Al_2_O_3_–MWCNTs	90	-	[44]
Commercial activated carbon	74	74	[45]
BI@MWCNTs	100	98	This work

## Data Availability

The raw/processed data generated in this work are available upon request from the corresponding author.
